# Effects of Balance Shoes on Balance and Postural Stability in the Elderly: A Crossover, Controlled, Randomized Single-Blind Study

**DOI:** 10.3390/healthcare9020179

**Published:** 2021-02-08

**Authors:** Nicolas Amiez, Carole Cometti, Éric Mouillon, Marie José Teisseire, Pascal Chenut, Christos Paizis, Nicolas Babault

**Affiliations:** 1 Center for Performance Expertise, CAPS, U1093 INSERM, Faculty of Sport Sciences, University of Bourgogne-Franche-Comté, 21000 Dijon CEDEX, France; amiez.nicolas@gmail.com (N.A.); carole.cometti@u-bourgogne.fr (C.C.); christos.paizis@u-bourgogne.fr (C.P.); 2 Axis Comfort Development, 81 Avenue des Bourdettes, 31250 Revel, France; eric.m@axisconfort.com (É.M.); mjteisseire@gmail.com (M.J.T.); pascalchenut21@gmail.com (P.C.)

**Keywords:** public health, technical assistance, falls, aging, postural balance, center of pressure, stability

## Abstract

The risk of falling increases with age. Individuals wearing unadapted shoes present an aggravating risk factor. The objective of this study was to determine the effectiveness of specifically designed balance shoes on balance and postural stability in healthy elderly people compared to that of their usual shoes. In total, 21 healthy individuals aged 65–84 years (76.0 ± 8.0 years) performed balance tests (bipedal with open or closed eyes, unipedal with open eyes, limits of stability, and step cadence) while wearing their (i) personal shoes or (ii) balance shoes (Axis Comfort Development©). Three test sessions were conducted with personal and balance shoes. The first served as the baseline, and the other two were performed after a familiarization period of several days with the personal or balance shoes. The perception of balance shoe efficiency was documented using a questionnaire. The balance shoes significantly improved bipedal balance with closed eyes. Moreover, the familiarization period significantly improved unipedal balance with open eyes. Most subjects felt safer and stabler using balance shoes. The investigated specifically designed balance shoes were effective in elderly individuals in improving postural balance compared to personal shoes. The balance shoes could, therefore, reduce the falling risk in healthy elderly people.

## 1. Introduction

Many falls experienced by older people result from age-related deterioration of the balance, concomitant with the neuromuscular system. More than 30% of people over 65 years old fall each year, with half of the falls occurring at home [1]. Falls represent 90% of hip fractures, and sometimes cause long-lasting physical and psychological effects [2,3]. This increase in falling is likely linked to the diminished capacity of some subsystems, such as the muscular (e.g., sarcopenia [4]) and somatosensory systems, cognition, or even individual behavior [5]. Many elements were identified as potential risk factors in the elderly, such as foot pain, chronic disease, high body mass index, a sedentary lifestyle, or wearing unstable shoes such as high heels [6,7,8].

Shoes are the direct link between feet and the ground, and they play a major role in postural control. There is a link between shoes, feet, and daily activity in the risk of falling [9]. If inappropriately designed, they can decrease postural stability [10] and modify the walking pattern [11]. However, evidence demonstrated that 30% of the elderly are barefoot or only wear socks at home [12,13]. Although being barefoot appeared to enhance sensory feedback and thus decrease instability compared to inappropriate shoes, such as sandals [10], it also increased the subjective perception of instability while walking [1], and was demonstrated to be a risk factor of falling for elderly people [14]. Shoes are, therefore, recommended. However, the shoes of elderly people are often considered hazardous for multiple reasons. For example, aesthetics is often the first selection criterion for women [7], and shoes worn at home are chosen for their low cost and comfort [13]. In addition, more than 60% of old people wear unadapted shoes, with inappropriate length and width that may cause pain and injury to the feet [15]. Therefore, only 1 in 9 elderly people wear fitted shoes at home [12]. To limit the risks of falls, shoes should present characteristics such as low and bevel-edged heels, being ankle boots with optimal heel support, nonslip, and with soles designed to facilitate proprioceptive feedback. For example, the width [7] and rigidity of the sole [16] were shown to improve postural balance [17].

For that purpose, balance shoes with increased width and thereby ground support surface were developed to prevent the risk of falls. Therefore, the primary aim of this study was to confirm the hypothesis that the type of sole of specifically designed balance shoes would improve balance in elderly people as compared to daily personal shoes. The secondary aim was to determine whether the effects of such shoes are immediate or require a familiarization period. We hypothesized that balance shoes are efficient for the balance of elderly individuals, and that a short familiarization period is recommended to optimize likely positive effects.

## 2. Materials and Methods

### 2.1. Study Design

In this crossover, controlled, randomized single-blind trial, healthy elderly individuals were tested on 3 separate occasions (baseline, control, and experimental test sessions) to test the effects of specifically designed shoes on balance. The control and experimental sessions were randomly presented. Familiarization periods lasting several days were used between two test sessions to determine the delayed effects of the shoes. Balance was evaluated on a posture platform and consisted in bipedal balance with eyes opened or closed, unipedal balance, limits of stability test, step cadence test. Subjective perception of the balance shoes was also addressed using a questionnaire.

### 2.2. Participants

Sample size was a priori determined using G*Power 3.1. The following parameters were used: α error = 0.05, power = 0.9, and effect size = 0.8. The estimated sample was 15. This sample was increased to compensate for possible volunteer losses. In total, 21 healthy volunteers were recruited for this study (18 women and 3 men). The anthropometric characteristics of the volunteers are presented in Table 1. All included volunteers were heathy, able to walk without any external help, and autonomous during their daily life. Volunteers were recruited from different local elderly organizations or personal connections. Inclusion criteria were: age between 65 and 85 years old, being able to stand on 1 foot for more than 10 s, and being able to walk and to stand up without any external help and without any significant pain. Volunteers were not included in cases of psychological, cardiological, pneumological, neurological, or osteoarticular disorders. Before participation, participants were fully informed of the purpose of the study and experimental procedure. A self-completion questionnaire was requested that included basic, demographic, medical treatment, and medical-history data. All subjects gave their informed consent for inclusion before they participated in the study. The study was conducted in accordance with the Declaration of Helsinki, and the protocol was approved by the local human research committee. All volunteers signed an informed-consent form. Approval was obtained from a local human research committee (ethics committee for STAPS research; CERSTAPS, IRB00012476-2020-24-03-48). The study was registered at ClinicalTrials.gov (NCT04344223). Throughout the study, subjects were asked to maintain a normal lifestyle.

### 2.3. Experiment Procedure

**Figure 1:**
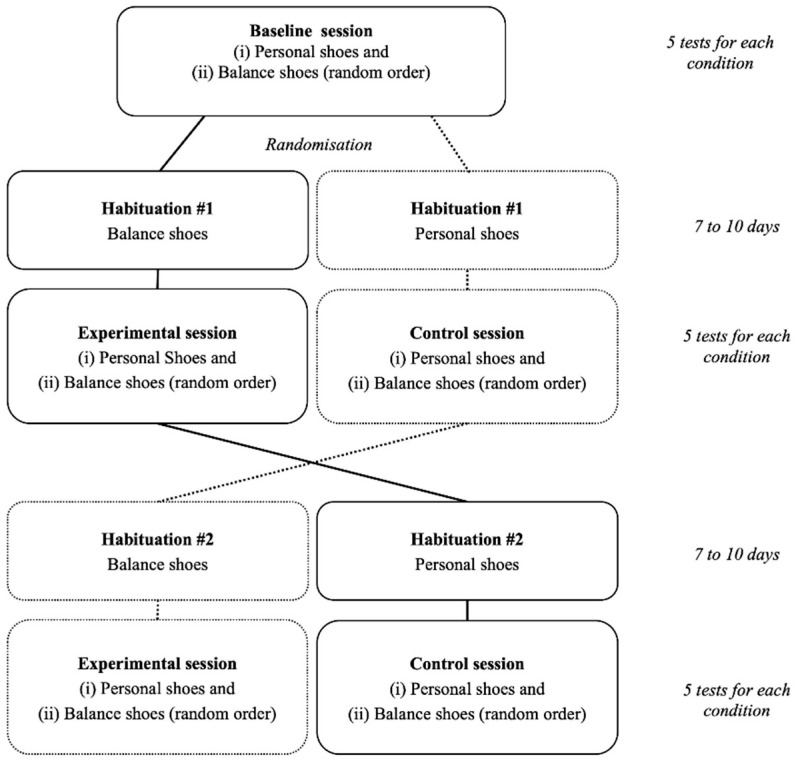
Experiment design.

Participants carried out 3 similar test sessions separated by 2 habituation periods lasting 7 to 10 days (Figure 1) to explore the effects of balance shoes on balance and postural stability. Test sessions were always performed at the same hour of the day for a given participant to limit any bias resulting from circadian rhythm effects. Room temperature and humidity were kept constant during all test sessions. Moreover, volunteers were requested to maintain their daily life habits during the entire experiment (activity, nutrition, hydration) and to limit extra activity that could cause unexpected fatigue. Balance shoes were developed by Axis Comfort Development© (Revel, France). The specific design of the shoes can be found on the following website: https://www.balance-shoes.com/en (accessed on 8 February 2021). Balance shoes were compared with personal shoes and chosen by the volunteers. Personal shoes corresponded to their outdoor shoes usually worn during daily activities within the last 2 months.

Each of the 3 test sessions included 2 test series of 5 different tests randomly presented. Each test series was carried out with a given type of shoes from the 2 being tested: (i) personal (Personal) or (ii) balance (Balance) shoes. The type of shoes was randomly presented. Before each test series, participants were shortly familiarized with the shoes being tested (3 min walk) followed by 2 min of sitting while instructions for the different tests were repeated. Fifteen minutes of rest were permitted between the two-test series.

The first test session served as the baseline. The 2 others explored the effects of personal of balance shoes after a 7-to-10-day habituation period. The experimental test session followed a habituation period with balance shoes. The other test session followed a habituation period with personal (daily) shoes and served as the control. During the habituation periods, participants were asked to wear shoes on a daily basis, being active for at least 1 h per day. Only for the habituation period performed while wearing balance shoes, participants completed a daily log aiming at reporting the approximate daily duration of wearing the balance shoes. Volunteers wore balance shoes on average for 276 ± 175 min per day. Large between-individual variability was obtained because volunteers were free to wear shoes as they wanted. Moreover, the daily duration was consistent between days.

### 2.4. Measurements

**Figure 2:**
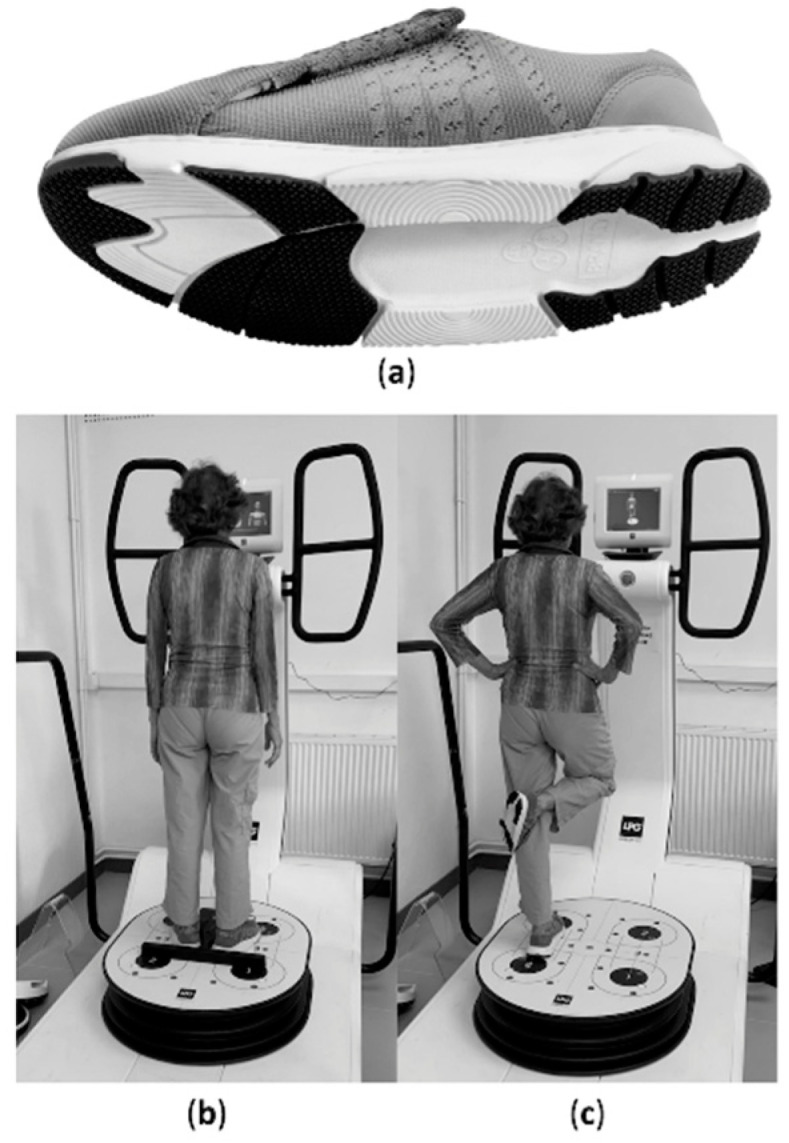
(**a**) Sole of balance shoes under investigation; (**b**) bipedal balance test on posture platform; (**c**) unipedal balance test on posture platform.

All tests were conducted on a Huber 360© balance and posture platform (LPG System, Valence, France; Figure 2). This platform was used as it permitted to measure the position of the center of pressure (CoP) and contacts with the ground during multiple steps. Each test was separated by a 1 min rest period. Tests included bipedal balance with eyes opened, bipedal balance with eyes closed, unipedal balance, limits of stability, and a step cadence test (served to mimic walking).

Bipedal balance was evaluated with eyes opened and closed for 50 s. Participants stood upright, with legs extended with a standardized feet position on the platform according to foot bars. Hands were kept along the trunk. In the open-eye condition, subjects had to watch a screen point in front of them. During these 2 tests, the surface area (Area; mm^2^), total displacement (Length; mm), and average speed (Speed; mm·s^−1^) of the CoP displacement were measured.

Unipedal balance was evaluated with visual control for 30 s. Participants stood upright on the right foot with the right leg extended. The right foot was positioned in a standardized position (according to a foot bar). The left leg was flexed with a 90° knee angle with the left knee maintained in contact with the right knee. Participants were instructed to hold their hands on their hips and to watch a screen point in front of them. Recording started when volunteers achieved a stable position. The surface area (Area; mm^2^) and total displacement (Length; mm) of CoP displacement were measured. During the 3 balance tests, in the case of an error (incorrect position or fall), the test was repeated.

The limits of the stability test aimed to quantify the maximal distance that the participant could achieve while maintaining their stability in 8 directions (every 45° intervals). Participants stood upright with legs extended. They had to maximally incline in a given direction while following a randomly presented target on the screen. Subjects were free to use their entire body to achieve the greatest possible displacement of their center of gravity from the initial position. Direct feedback was present on the platform screen to help volunteers to shift their center of gravity in accordance with the imposed direction and give direct feedback on performance. The participants were instructed to keep their feet on the ground. Recordings lasted 8 s in each direction. The maximal distance travelled by the CoP in each direction was measured.

During the step-cadence test, volunteers had to mimic walking on a spot over the platform for 50 s. Knee rise had to be sufficient to bring the foot halfway up the support leg. The subject had to follow the cadence indicated on the screen (93 steps in total). The platform measured the number of steps.

In addition, the subjective perception of the balance shoes was documented using a structured questionnaire at the end of the habituation period of the experiment session. The questionnaire was composed of 4 closed questions related to the perception of stability, safety, comfort, and aesthetics. Questions were (a) “How do you feel in general wearing balance shoes?” (very comfortable; comfortable; normal; uncomfortable); (b) “How do you find the aesthetics of balance shoes?” (nice; correct; ugly; no opinion); (c) “Did you feel stabler wearing balance shoes?” (yes or no); and (d) “Did you feel safer wearing balance shoes?” (yes or no). Volunteers were instructed to respond while comparing balance shoes to their personal shoes.

### 2.5. Statistical Analysis

Data analysis and statistics were conducted blinded. Mean values and 95% confidence intervals are presented. After verifying the application conditions (sphericity and normality of distributions using Mauchly and Kolmogorov–Smirnov tests, respectively), 2-way analysis of variance (ANOVA) with repeated measures was used (Sessions × Shoes). Sessions corresponded to the different test sessions: baseline vs. experimental vs. control. Shoes corresponded to tests performed using balance or personal shoes. F ratios were considered significant at *p* level < 0.05. In the case of significant main effects (Session or Shoes) or significant interaction (Session × Shoes), a post hoc test using Bonferroni correction was carried out to study the specific differences. Statistical analysis was performed using Statistica© ver 7.0 (Statsoft, Tulsa, OK, USA) software.

## 3. Results

**Figure 3:**
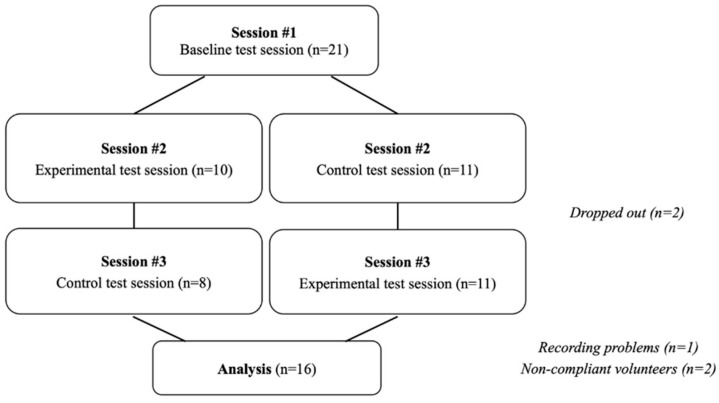
Study flowchart.

After completion of the experimental procedure, 16 individuals (13 women and 3 men) were considered for statistical analysis (see CONSORT flowchart in Figure 3).

Results of the repeated-measure analysis of variance and data are presented in Table 2 and Table 3, respectively. As indicated, significant main effects were obtained for (i) the CoP Area during the bipedal balance test eyes closed (Shoes factor), (ii) the CoP Length during unipedal balance test (Session factor) and (iii) 315° stability limit (Shoes factor). Post hoc analysis performed on the bipedal balance (eyes closed) revealed a significantly smaller rea with the balance shoes as compared to that with the personal shoes (*p* = 0.0475). Post hoc analysis conducted on unipedal balance revealed significantly smaller CoP length with the balance shoes during both the experimental and control sessions as compared to that of the baseline (*p* = 0.0319 and *p* = 0.0151, respectively). Lastly, the limit of stability was lower at 315° with balance shoes as compared to that with the personal shoes (*p* = 0.0165).

The subjective perception of the balance shoes is shown in Table 4. All volunteers found that the balance shoes were “comfortable” or “very comfortable”. Thirteen volunteers found that they looked good. Lastly, the majority found that stability and safety were improved with the balance shoes.

## 4. Discussion

Our findings demonstrated that the investigated balance shoes (specifically designed for stability, i.e., with increased ground surface) enhanced balance during the most difficult conditions in healthy elderly individuals. Specifically, balance was enhanced in the unstable condition (bipedal with closed eyes). Moreover, because some differences were observed between the baseline test session and the two others, we concluded that the balance shoes’ effects were optimized after a short familiarization period (here, between 7 and 10 days). Lastly, volunteers perceived higher stability and safety with the balance shoes as compared to with their daily personal shoes.

Aging reduces the efficiency of the sensorimotor and neuromuscular systems, which deteriorates balance. The control of postural reactions becomes slower and disorderly. In addition, postural control decreases in the frontal plane. Therefore, balance requires increasing attention. The fear of falling is also more present with significant psychological repercussions [18]. These changes can be quantified by a faster and longer displacement speed of the CoP during the bipedal station with eyes open and closed [19]. Moreover, the majority of elderly individuals paid little attention to the health of their feet and were using footwear of the incorrect size, a circumstance that corresponded with a lack of foot sensitivity, and the appearance of a variety of foot conditions and deformities [20]. Therefore, intervening directly on the only link with the ground using adequately fitted shoes seems to be a viable solution to use concurrently with physical activity to improve balance and reduce the risk of falling.

As expected, the balance shoes used here enhanced postural stability in the most difficult conditions. Similar results were previously obtained when comparing classical slippers to a pair combining a set of key criteria to improve balance (rigid soles, closed heels, easy tightening). Despite the different shoe characteristics, only a walk test made it possible to differentiate the two shoe types [21].

Unipedal balance is a complex task that is important for all our daily activities (e.g., while walking). It is characterized by two phases: the first, so-called dynamic, phase corresponds to the transfer of the body weight on one side; the second, so-called static, phase is when balance is found [22]. The dynamic phase could be influenced by age-related neuromuscular weakness and the visual field, and the distance of the feet preceding the transitional period between bipedal and unipedal stations can also influence performance [23,24]. For these reasons, we measured the unipedal static phase while fixating the gaze to limit unexpected variations. We observed improvement in unipedal balance following the first habituation period for both the experimental and the control session only for the balance shoes. Accordingly, becoming familiar with the shoes was of paramount importance to observe significant effects. Familiarization was, therefore, persistent even if personal or other shoes were frequently used.

Importantly, the unipedal balance in the frontal plane becomes precarious with aging, and increases the risks of lateral falls and their severity [23]. However, balance in the frontal plane seems to be based on common motor patterns to the sagittal plane. The motor control of balance would, therefore, be based on an ecological strategy with preferential recruitment of the flexor and extensor muscles of the foot to respond to disturbances [25]. These results were supported a few years later, proving the major role of plantar flexors in the displacement of the center of pressure in the mediolateral axis [26]. Mediolateral sway is an important clinical parameter because it increases with age more than anteroposterior sway does [27], and it is greater in fallers than in age-matched nonfallers [3]. Moreover, footwear may optimize the biomechanical alignment of the lower limbs, thus promoting the efficiency of postural control [28,29]. Therefore, increasing the width of the sole of the shoes (as with the balance shoes here) increases the support surface in the frontal plane but also helps to maintain balance in all directions.

Balance is influenced by the somatosensory system, but is also under visual supervision. The voluntary suppression of vision during a postural-balance task considerably increases the difficulty. Visual information is important to maintain balance, especially when proprioceptive afferents are of low quality [30]. Thus, suppressing the vision constrains individuals to use mainly auditory, vestibular, and proprioceptive feedback, which are less effective with aging. Balance shoes, therefore, are an important asset to improve balance, as shown by the reduction in the area covered by the CoP during the bipedal task with eyes closed. Other results confirmed the importance of wearing shoes on postural balance, even in young athletes [31]. However, wearing balance shoes is not the only strategy that could be used to improve balance in the elderly. For instance, a recent study showed that hearing amplification could be helpful in improving stability [32].

Our results revealed the positive effects of balance shoes during unstable tasks. Other tasks were not influenced by stabilization shoes. Indeed, we were unable to observe any change in the limits of stability in most directions. The obtained result for a single angle (i.e., 315°) was in favor of personal shoes. This exception could be attributed to large inter-volunteer variability and potential laterality effects. Further analyses should be conducted to explain this surprising angle-specific finding. However, the general lack of effect regarding the limits of stability was already mentioned by other authors who compared modified slippers to barefoot and classical slippers conditions [1]. Other authors measured the effect of wearing unstable shoes for several weeks on postural balance and observed deterioration in performance on several tests without impacting the test of the stability limits [33].

Being aware of the beneficial effects of adapted shoes for health is not sufficient to convince elderly people to leave their daily shoes. The comfort of a shoe is subjective and not necessarily linked to biomechanics [34]. It is an important criterion when buying shoes for home, but becomes of secondary importance for women when buying shoes for outdoors [7,13]. This choice is explained by the social standards imposed on women [35]. Therefore, the aesthetic criterion is essential for shoes to be accepted and worn by the elderly both at home and outdoors [7]. In addition, suffering from foot pain causes a decrease in balance and negatively impacts the activities of daily life on physical and psychological plans [36,37]. For example, adapted wider shoes are recommended by clinicians to limit the pain caused by some diseases, such as claw or hammer toes, which could degrade balance [38]. Thus, the generally positive responses to our questionnaire concerning the balance shoes permitted to assume a positive and strong enrolment to these shoes; thus, they guarantee a real effect on reducing the risk of falls both at home and outside.

According to our inclusion criteria, the conclusions of the study cannot be generalized to volunteers with intrinsic instability. Moreover, some confounding factors such as gender, physical activity, anthropometry, foot morphology, and auditory and vestibular deficiency could have influenced our findings and should be tested in future studies. Only some tests (standardized and well-documented in the literature) were used for the present experiment. Other tests in fresh or fatiguing situations could be of interest. Volunteers self-reported the use of balance during the habituation phases. The total time during which volunteers wore the balance shoes tested here was inevitably different, but agreed with our experiment instructions and criteria. Moreover, volunteers were allowed to wear their personal shoes in alternance with the balance shoes. A ratio between time with balance vs. personal shoes was of interest but was impossible to calculate. Lastly, the present study compares balance shoes with daily personal shoes. Different shoe types and designs were, therefore, compared with the balance shoes. Such a comparison strengthens the present conclusion and is also a methodological limitation.

## 5. Conclusions

The balance shoes here (specifically designed to increase balance) improved postural balance in some situations, particularly in the most difficult conditions (bipedal with eyes closed). The specifically designed shoes helped the somatosensory system when vision was suppressed. Wearing shoes during a short familiarization period (here, between 7 and 10 days) is necessary to optimize the resulting effects. Moreover, the majority of the participants reported positive effects of balance shoes as compared to their daily personal shoes. The balance shoes tested here can, therefore, be a solution to improve the safety of elderly people and prevent falls.

## Figures and Tables

**Table 1:** Anthropometric characteristics of study volunteers.

Variable	Women (*n* = 18)	Men (*n* = 3)	All (*n* = 21)
Age (years)	71.4 (69.2–73.7)	76.0 (51.5–100.4)	72.0 (69.6–74.5)
Height (cm)	162.7 (159.8–165.7)	171.3 (152.4–190.3)	164.0 (160.9–167.1)
Body mass (kg)	62.9 (58.4–67.4)	76.0 (47.3–104.6)	64.7 (60.1–69.4)

Values are numbers (*n*) or mean (95% confidence interval).

**Table 2:** Results of repeated measure analysis of variance (*p* values).

Test	Parameter	Session	Shoes	Session × Shoes
Balance on two feet, eyes open	Length	0.4918	0.5558	0.9236
	Area	0.2018	0.4855	0.4392
	Speed	0.5049	0.5422	0.9192
Balance on two feet, eyes closed	Length	0.2273	0.5594	0.4716
	Area	0.9126	0.0475 *	0.6508
	Speed	0.2272	0.5599	0.4720
Balance on one foot	Length	0.0292 *	0.7218	0.1056
	Area	0.2453	0.2993	0.3647
Stability limits	0°	0.4195	0.0708	0.3806
	45°	0.8125	0.4916	0.7770
	90°	0.1795	0.5650	0.0916
	135°	0.4976	0.5976	0.4972
	180°	0.3020	0.9078	0.5632
	225°	0.4011	0.6305	0.6238
	270°	0.9149	0.6367	0.2476
	315°	0.2692	0.0165 *	0.7599
Walking	Steps	0.3210	0.1481	0.9274

* significant main effect or interaction (*p* < 0.05).

**Table 3:** Obtained data during different test sessions while wearing balance or personal shoes.

Test	Index	Baseline		Experimental		Control	
		Personal	Balance	Personal	Balance	Personal	Balance
Balance on two feet, eyes open	Length (mm)	667 (574–761)	685 (606–764)	697 (620–774)	713 (625–801)	686 (610–761)	687 (598–776)
	Area (mm^2^)	223 (153–292)	240 (176–303)	296 (197–395)	252 (191–312)	275 (181–370)	250 (179–321)
	Speed (mm/s)	13.4 (11.5–15.3)	13.7 (12.2–15.3)	13.9 (12.4–15.4)	14.3 (12.5–16.1)	13.7 (12.2–15.2)	13.7 (11.9–15.5)
Balance on two feet, eyes closed	Length (mm)	1442 (1010–1874)	1496 (995–1996)	1365 (977–1752)	1290 (1036–1543)	1458 (1033–1882)	1377 (1057–1696)
	Area (mm^2^)	739 (516–962)	702 * (383–1020)	786 (525–1048)	629 * (455–804)	796 (526–1066)	678 * (444–912)
	Speed (mm/s)	28.8 (20.1–37.4)	29.9 (19.9–39.9)	27.3 (19.5–35.1)	25.8 (20.7–30.9)	29.2 (20.7–37.7)	27.6 (21.2–34.0)
Balance on one foot	Length (mm)	1892 (1535–2248)	2022 (1602–2441)	1851 (1538–2163)	1717 (1413–2020)	1798 (1501–2094)	1756 $ (1516–1995)
	Area (mm^2^)	960 (426–1493)	2435 (−739 to 5609)	771 (641–900)	839 (673–1004)	962 (432–1492)	774 (595–952)
Limits of stability	0°	112.4 (102.9–121.9)	102.4 (94–110.8)	111.1 (102–120.2)	102.2 (95.6–108.8)	109.9 (102.3–117.5)	110.3 (101.3–119.3)
	45°	150.3 (128.1–172.5)	153.8 (136.9–170.7)	148.3 (135.8–160.8)	147.9 (136.5–159.3)	147.5 (139.8–155.2)	154.5 (142.3–166.7)
	90°	189.1 (175.5–202.7)	184.6 (170–199.2)	181.9 (167–196.8)	189.1 (173.6–204.6)	189.9 (175.8–204)	193.4 (178.9–207.9)
	135°	151.9 (139.2–164.6)	159.6 (147.5–171.7)	151.8 (135.8–167.8)	157.8 (143.2–172.4)	150.2 (128.5–171.9)	154.1 (142.5–165.7)
	180°	112.7 (102.5–122.9)	112.1 (101.9–122.3)	112.2 (99.9–124.5)	116.1 (106.8–125.4)	110.9 (99.8–122)	106.4 (96.3–116.5)
	225°	160.1 (144.9–175.3)	160.1 (141.5–178.7)	152.6 (135.6–169.6)	161.8 (144–179.6)	154.1 (135.7–172.5)	152.4 (140.7–164.1)
	270°	205.9 (196.3–215.5)	202.2 (190.1–214.3)	199.0 (181.3–216.7)	206.6 (196.6–216.6)	202.6 (191.5–213.7)	202.6 (189.6–215.6)
	315°	161.8 (152.6–171) *	146.8 (133.5–160.1)	153.3 (138.1–168.5) *	140.8 (125.8–155.8)	157.3 (147.4–167.2) *	149.4 (135.4–163.4)
Walking	Steps	84.6 (78.5–90.7)	86.6 (79.9–93.3)	87.7 (81.2–94.2)	89.6 (84.0–95.2)	88.1 (81.3–94.9)	89.1 (83.7–94.5)

Values are means (95% confidence interval). $, significant differences between baseline for same shoes (*p* < 0.05). *, significant differences between shoes for same session (*p* < 0.05).

**Table 4:** Subjective perception of balance shoes.

**How Do You Feel in General Wearing Balance Shoes?**	**Very Comfortable**	**Comfortable**	**Normal**	**Uncomfortable**
Responses	*n* = 8 (50%)	*n* = 8 (50%)	*n* = 0 (0%)	*n* = 0 (0%)
**How Do You Find the Aesthetics of Balance Shoes?**	**Nice**	**Correct**	**Ugly**	**No Opinion**
Responses	*n* = 1 (6%)	*n* = 13 (81%)	*n* = 2 (13%)	*n* = 0 (0%)
**Did You Feel More Stable Wearing Balance Shoes?**	**Yes**		**No**	
Responses	*n* = 15 (94%)		*n* = 1 (6%)	
**Did You Feel Safer Wearing Balance Shoes?**	**Yes**		**No**	
Responses	*n* = 12 (75%)		*n* = 4 (25%)	

Participant numbers and percentages are given.

## Data Availability

Data are freely available at http://dx.doi.org/10.17632/cwyvmmtxsg.1.
